# Electrical Stimulation Improves Rat Muscle Dysfunction Caused by Chronic Intermittent Hypoxia-Hypercapnia via Regulation of miRNA-Related Signaling Pathways

**DOI:** 10.1371/journal.pone.0152525

**Published:** 2016-03-29

**Authors:** Lu-Lu Pan, Jiang-Qiong Ke, Cui-Cui Zhao, Shi-Yuan Huang, Jie Shen, Xian-Xun Jiang, Xiao-Tong Wang

**Affiliations:** 1 Center of Neurology and Rehabilitation, the Second Affiliated Hospital of Wenzhou Medical University, Wenzhou, China; 2 Department of Rehabilitation, the Traditional Chinese Medical Hospital of Wenzhou City, Wenzhou, China; 3 Wenzhou Medical University, Wenzhou, China; University of Louisville School of Medicine, UNITED STATES

## Abstract

Skeletal muscle dysfunction in chronic obstructive pulmonary disease (COPD) patients is common. Neuromuscular Electrical Stimulation (NMES) is a powerful exercise training that may relieve muscle dysfunction in COPD. This study investigated whether electrical stimulation may have atypical adaptations via activation of miRNA related pathways in counteracting COPD muscle dysfunction. Forty-eight male Sprague-Dawley rats were randomly assigned to 3 groups. With the exception of the rats in the control group, the experimental rats were exposed to chronic intermittent hypoxia-hypercapnia (CIHH) (9∼11%O_2_,5.5∼6.5%CO_2_) for 2 or 4 weeks. Electrical stimulation was performed immediately after each CIHH session. Following assessment of the running capacity, biopsy samples were obtained from the gastrocnemius of the rats. The miR-1, miR-133a and miR-133b levels were measured, as well as their related proteins: phosphorylation of Akt (p-AKT), PGC-1alpha (PGC-1α), histone deacetylase 4 (HDAC4) and serum response factor (SRF). Myosin heavy chainⅡa (MHCⅡa) and myosin heavy chainⅡb (MHCⅡb) were also measured to assess fiber type changes. After 2 weeks, compared with the controls, only miR-1 and miR-133a were significantly increased (p<0.05) in the exposure group. After 4 weeks, the exposure group exhibited a decreased running distance (p = 0.054) and MHCⅡa-to-MHCⅡb shift (p<0.05). PGC-1α (p = 0.051), nuclear HDAC4 (p = 0.058), HDAC4, p-AKT, PGC-1α and SRF was also significantly decreased (p<0.05). In contrast, miR-1 and miR-133a were significantly increased (p<0.05). Four weeks of electrical stimulation can partly reversed those changes, and miR-133b exhibited a transient increase after 2 weeks electrical stimulation. Our study indicate miRNAs may have roles in the response of CIHH-impaired muscle to changes during electrical stimulation.

## Introduction

Chronic obstructive pulmonary disease (COPD) is a worldwide disease. Skeletal muscle dysfunction is common in COPD patients[1, 2]. In recent years, several studies have demonstrated that skeletal muscle dysfunction is associated with low exercise tolerance and a reduction in quality of life and may predict poor prognosis[3–5]. The major reason for muscle dysfunction may be I-to-II muscle fiber type shift and muscle atrophy, which lead to weakness and reduced endurance capacity in patients with COPD [6, 7]. And the complicated pathogenesis under muscle dysfunction remains elusive[3]. Chronic intermittent hypoxia hypercapnia (CIHH) is an animal model with demonstrated contributions to the understanding of the development of COPD pathophysiology[8–10]. In our previous study, exposure to CIHH deteriorated the muscles of the experimental rats when the exposure time was prolonged more than 2 weeks[9].

microRNAs (miRNAs) regulate many cellular processes by gene silencing via translational repression or target degradation [11]. MyomiRs represent a suite of miRNAs that are highly enriched in cardiac and/or skeletal muscle which including miR-1, miR-133a, miR-133b and so on[12]. They play important roles in the regulation of muscle development, growth, regeneration and metabolism through related proteins. miR-1 promotes muscle differentiation by targeting histone deacetylase 4 (HDAC4), a transcriptional repressor of muscle gene expression, and PGC-1alpha (PGC-1α); it also controls the muscle cell phenotype via the regulation of insulin-like growth factor 1 (IGF-1); in contrast, miR-133 modulates muscle proliferation via the repression of serum response factor (SRF)[13, 14]. An excellent study demonstrated that the IGF-1, SRF and HDAC4 signaling pathways, which are related to miR-1, have roles in muscle dysfunction in COPD patients[5].

Neuromuscular Electrical Stimulation (NMES) is a localized training strategy that is better suited to provide an adequate training stimulus while minimizing the impact on both the ventilatory system and cardiac requirements during exercise training[15], which is useful for strengthening peripheral muscles in patients with COPD[16, 17]. Recently, an interesting study has indicated that an appropriate training period and pattern associated with NMES may induce the atypical adaptations of the muscle. According to this research, NMES is characterized by both resistance (i.e., strength gains) and endurance (i.e., fast-to-slow and glycolytic-to-oxidative conversion) training[18]. We hypothesized that electrical stimulation may have atypical adaptations via activation of the miRNA-related signaling pathway in counteracting COPD muscle dysfunction. We therefore analyzed muscle-specific miRNA expression, the expression of miR-1 (an miRNA associated with IGF-1, HDAC4 and PGC-1α in skeletal muscle) and miR-133a/b (SRF-dependent miRNAs).

## Materials and Methods

### Ethics statement

All animal handling procedures and experimental protocols were approved by the Ethics Committee of Wenzhou Medical University(Approved No.wydw2014-0006). In accordance with the ethical guidelines for animal research, all efforts were made to reduce the number of animals and minimize their suffering.

### Animals

Forty-eight male, Sprague-Dawley rats, which weighted 160–180 g, were purchased from the Laboratory Animal Center of Wenzhou Medical University. The rats were housed under specific pathogen-free (SPF) conditions with a 12/12 h dark/light cycle at a 23±1°C room temperature. Food and water were available at discretion. The rats were acclimated to the laboratory conditions for at least 7 days prior to beginning the experiments.

### Experimental design

Rats were selected based on similar endurance running capacities (subsequently described) and then randomly assigned to 3 groups (16 per group): (i) normal control group (NC); (ii) hypoxia-hypercapnia group (HH); and (iii) hypoxia-hypercapnia + electrical stimulation group (HE), then each group was divided into 2 weeks group and 4 weeks group (8 per group). The CIHH model was induced as previously described[10]. In brief, the rats in the HH and HE groups were intermittently placed in a closed chamber for 8 h/day (usually from 8am to 4pm) and maintained in a CO_2_ gas mixture environment (9%–11%O_2_+6.5%–7.5%CO_2_ in N_2_). The exposure cycle was performed 7 days/week for 2 or 4 weeks. The NC group was placed in a closed chamber with the same conditions as the experimental groups, with the exception that the CO_2_ gas mixture environment was replaced with a normal air environment. Electrical stimulation was performed immediately after the first CIHH session and until the last day. The rats in all groups were maintained in an immobilization apparatus as previously described[19] during each electrical stimulation session; however, only the HE group underwent electrical stimulation using an apparatus (HANS-200E, Jisheng Medical Instruments), whereas the other groups only underwent immobilization. The total length of the experimental period was 4 weeks, which was followed by the assessment of endurance running capacity (subsequently described). Biopsy samples were subsequently obtained from the gastrocnemius of the rats.

### Electrical stimulation protocol

The rats were fixed in an immobilization apparatus, and two surface electrodes (1.5 cm×1.5 cm) were placed in the bilateral gastrocnemius after shaving. The muscles were subsequently stimulated for 30 min/day. The stimulus parameter: every 3-s pattern of 100 HZ stimulation followed by a 3-s pattern of 2 HZ stimulation (pulse duration:0.3~1.0 ms,according to frequency changes), the intensity (2–5 mA), was adjusted to induce moderate contractions.

### Estimation of endurance running capacity

The protocol used to estimate the aerobic running capacity was performed as previously described[20], with modifications. The first section consisted of introducing each rat to the treadmill (model ZH-PT,HuaiBei ZhengHua Biology Instruments) to gradually increase the duration each day. The goal of this section is to ensure that each rat could run at a speed of 10 m/min on a 15° slope. The ability to achieve this minimal level of running comprised the threshold performance used to assess the maximal running capacity in the subsequent days. First, the rats were placed on the belt (10 m/min, 15° slope) and continuously picked up and moved forward to prevent the rats from sliding off the back of the belt. Next, the belt speed was gradually increased to 15 m/min; when the rats failed to run and slide off of the belt, they fell onto a 15×15 cm electric shock grid that delivered 1.2 mA of current at 3 Hz. This process was subsequently repeated until the rats learned to run for 5 min to avoid the mild shock. This amount of exposure to treadmill running is not likely to produce a significant change in aerobic capacity[21]. During the following 2 days, the maximal endurance running capacity was determined. The endurance trial was initiated with a 10 m/min,15° slope, and the treadmill velocity was increased by 1 m/min every 2 min. Each rat ran until exhausted, and the total distance run (in meters) was used as the estimate of aerobic endurance capacity. Exhaustion was defined as the third time a rat could no longer maintain its pace with the speed of the treadmill and remained on the shock grid for 2 s rather than running. At the moment of exhaustion, the rat was removed from the treadmill, and the total distance run was recorded.

### Immunofluorescence

Muscle specimens were obtained from the gastrocnemius muscles of both legs and directly mounted in an OCT compound (Tissue OCT-Freeze Medium) (Tissue Tek, Miles Laboratories, Naperville, IL, USA) on a thin cardboard. The muscles were then rapidly frozen in liquid isopentane with liquid nitrogen. Serial 10-μm transverse sections were cut at –20°C, thaw mounted on slides and stored at –80°C until analysis. Staining was performed as previously described[5]; the sections were incubated at 4°C overnight with a mixture of primary antibodies in PBS with 0.05% Tween 20 (PBST) (rabbit anti-SRF,1:100,Santa Cruz). After washing the slides for five minutes in PBST three times, the sections were incubated with a secondary antibody mix in PBST (Goat anti-rabbit IgG-FITC,1:100,Santa Cruz) in the dark and a humidification box for one hour at room temperature. The slices were treated with diamino-phenylindole (DAPI) after washing the slides for five minutes in PBST two times. Finally, the slides were washed for five minutes in PBST and Faramount aqueous mounting medium (Dako, USA); a coverslip was applied, and the slides were stored in the dark at 4°C. The sections were mounted and examined using an Olympus FluoView FV500 confocal microscope (OLYMPUS AMERICA INC.2 Corporate Center Drive, Melville, NY 11747–3157, USA). Control sections were incubated with PBS instead of primary antibodies.

### Western blotting analysis

Western blotting was performed as previously described[10]. The blots were probed with mouse anti-PGC-1α (1:1000,Calbiochem), mouse anti-MHCⅡa (1:200,DSHB), mouse anti-MHCⅡb (1:200,DSHB), rabbit anti-p-AKT (1:2000,Cell Signaling), moues anti-HDAC4 (1:1000, Cell Signaling), and rabbit anti-SRF (1:200,Santa Cruz), and the secondary antibody was anti-rabbit or anti-mouse. Bands were visualized via chemiluminescence and quantified by densitometry. The proteins were subsequently visualized with BeyoECL Plus reagents and captured using an enhanced chemiluminescence system (ECL kit, Pierce Biotechnology, Inc).

### microRNA quantification

microRNA expression was analyzed in TRIzol extracted RNA according to the manufacturer’s instructions (the All-in-One™ miRNA qRT-PCR Detection Kit, GeneCopoeia). Forward primers specific for each miRNA were obtained from GeneCopoeia, and the reverse primer was present in the kit. The qPCR reactions were run on the Roche LightCycler 480 real-time PCR system (Roche Co,Germany) with the following cycle program: 95°C for 10 min, followed by 40 cycles of 95°C for 10 s, 60°C for 20 s, and 72°C for 10 s. The PCR products were run on a 2% agarose gel to confirm the correct base pair size. 5S RNA from the same sample was used as an endogenous control.

### Statistical analysis

The data were analyzed by one-way ANOVA followed by a post hoc comparison test using the LSD (equal variances assumed) or Dunnett’s T3 (equal variances not assumed) method. A value of p<0.05 was considered statistically significant. The data were expressed as the means ± SEMs. All statistical procedures were performed with SPSS16.0 software.

## Results

### 1. Changes in endurance running capacity and muscle phenotype

Aerobic capacity is important in many diseases, and changes in the running capacity partially reflect muscle impairments. To investigate if there were changes in the endurance running capacity, a motorized treadmill was used to determine the running distance. At the end of the experiment (after 4 weeks), the running distance of the HH group (406±40.2 m) was lower than the NC group (532±47.4 m,p = 0.054). Electrical stimulation (HE group) induced a significant improvement in the running distance (539±44.9 m) compared with the other groups (p<0.05).

**Fig 1:**
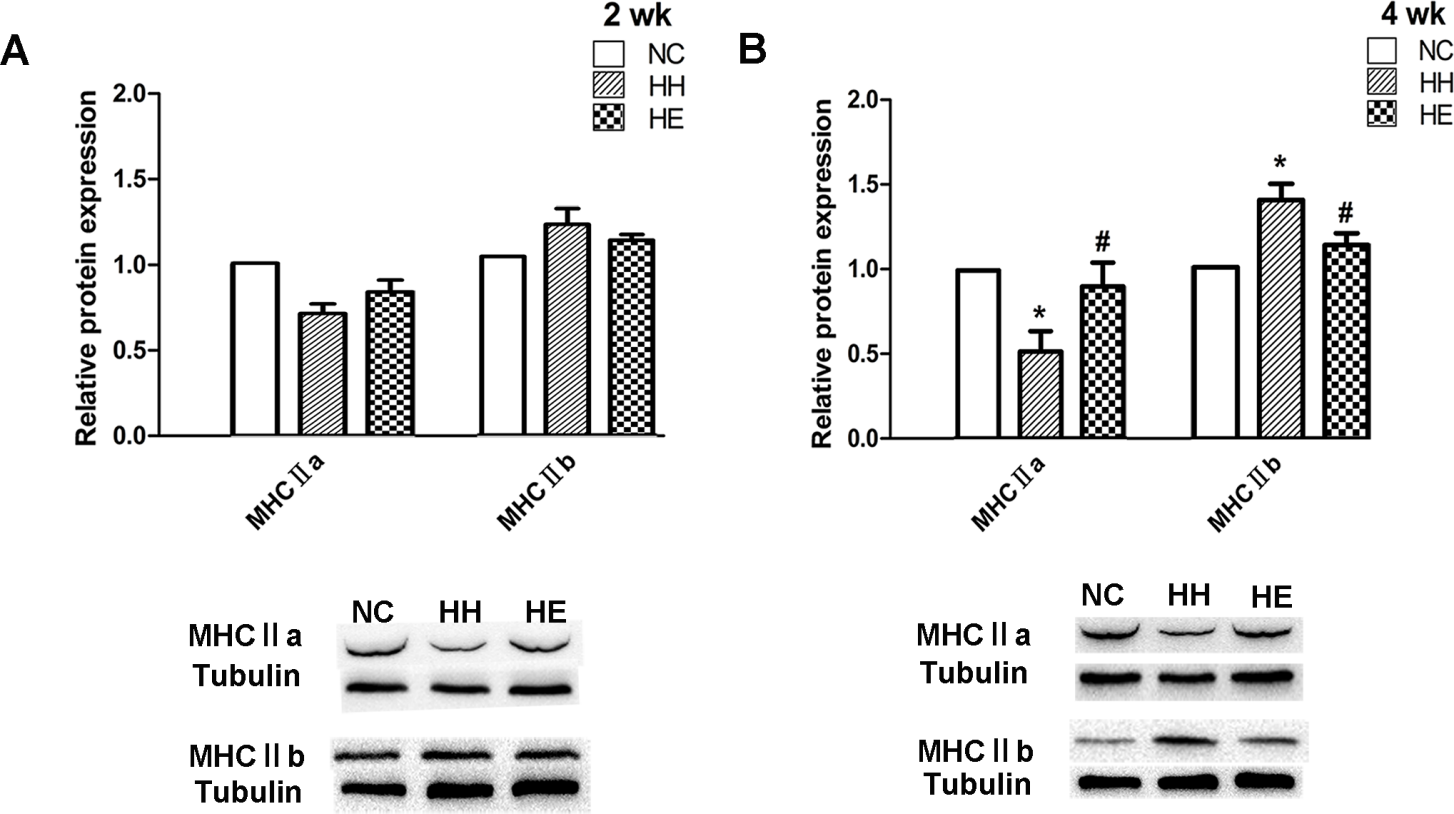
Effects of electrical stimulation on the CIHH-induced expression of the relative myosin heavy chain (MHC) protein content expression. Gastrocnemius muscles were isolated from the lower limb of three groups of rats at 2 weeks (A) and 4 weeks (B). Analyses of the expression of MHCⅡa and MHCⅡb were performed by Western blotting. Tubulin was used as a loading control. The optical density values were normalized to their respective Tubulin loading control, and the means±SEMs were graphed (relative expression) to semi-quantitatively compare the protein levels. *p<0.05 (at least) vs. NC group; #p<0.05 (at least) vs. HH group. NC = normal control group; HH = hypoxia-hypercapnia group; HE = hypoxia-hypercapnia + electrical stimulation.

To investigate changes in the oxidative potential in muscle fibers, Western blotting (Fig 1) was performed to determine the expression of myosin heavy chainⅡa (MHCⅡa) and myosin heavy chainⅡb (MHCⅡb) proteins. We regarded MHCⅡa as the slow MHC and MHCⅡb as the fast MHC because in rat muscles, the abundance of mitochondria and oxidative enzymes are highest in Ⅱa fibers and lowest in Ⅱb fibers, which are very different from human muscles[22, 23]. After 2 weeks (Fig 1A), the content of the slow MHC (MHCⅡa) in the HH group had a downward trend, whereas the fast MHC (MHCⅡb) in the HH group was increased; however, these changes were not significantly different among the three groups. Four weeks (Fig 1B) of CIHH exposure resulted in a significant decrease in MHCⅡa in the HH group compared with the NC group (p<0.05); in contrast, the content of MHCⅡb protein was significantly increased compared with the NC group (p<0.05), and electrical stimulation partially reversed these changes (p<0.05). Thus, there were muscle fiber type changes between the slow and fast fibers in the groups.

### 2. Changes in miRNAs expression

**Fig 2:**
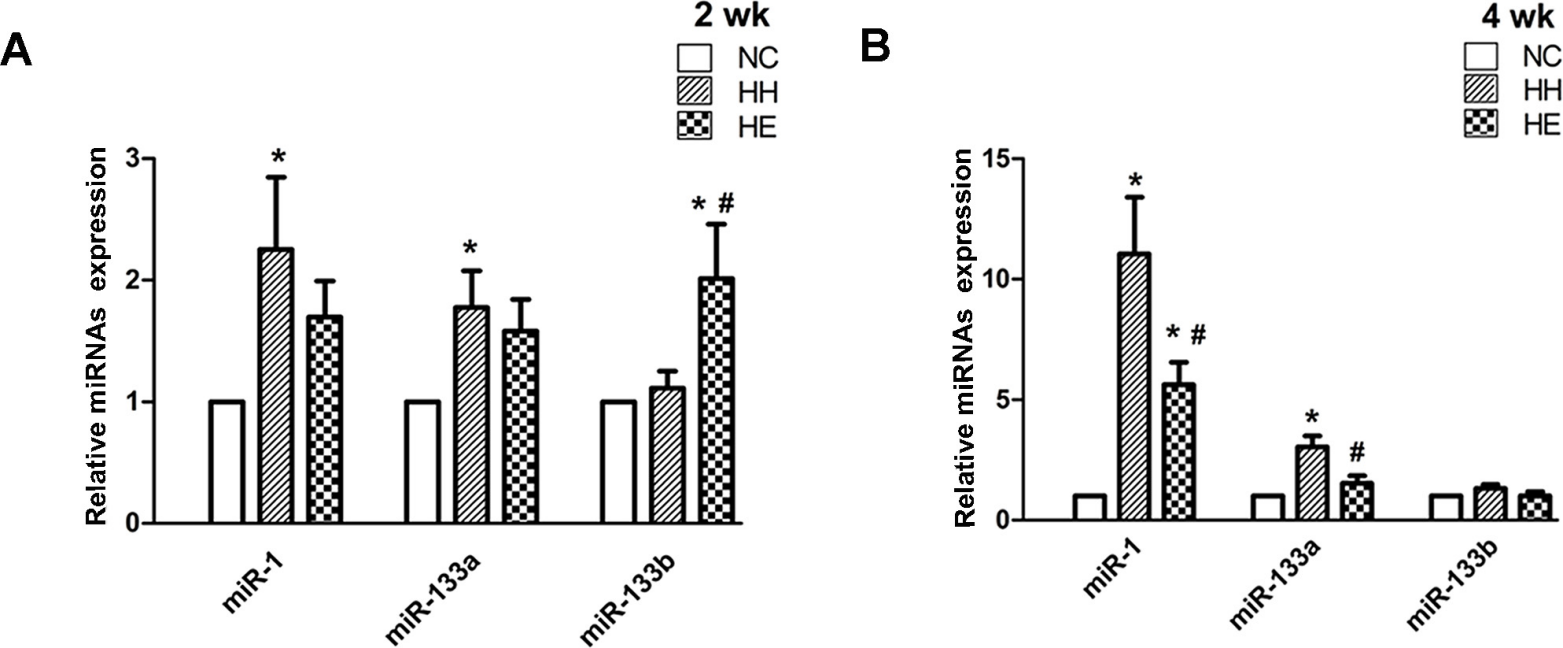
Effects of electrical stimulation on the CIHH-induced expression of miR-1, miR-133a and miR-133b. Gastrocnemius muscles were isolated from the lower limb of three groups of rats at 2 weeks (A) and 4 weeks (B). Analyses of the expression of miR-1, miR-133a and miR-133b were performed by qRT-PCR and normalized to the expression of 5S RNA in the same sample, as described in the Methods section. *p<0.05 (at least) vs. NC group; #p<0.05 (at least) vs. HH group. Data are expressed as the means ±SEMs. NC = normal control group; HH = hypoxia-hypercapnia group; HE = hypoxia-hypercapnia + electrical stimulation.

miR-1 and miR-133 have distinct roles in the modulation of skeletal muscle proliferation and differentiation[13]. Thus, we investigated miRNAs using qRT-PCR. At 2 weeks (Fig 2A), the relative miR-1 and miR-133a expression of the HH group were significantly increased compared with the NC group (p<0.05). However, the miR-133b expression was not significantly different between the groups. The HE group exhibited downward trends in the miR-1 and miR-133a expression compared with the HH group; but, these changes were not significant. In contrast, the miR-133b expression was substantially increased after 2 weeks of electrical stimulation compared with the HH group (p<0.05) and the NC group (p<0.05). Four weeks of CIHH exposure (Fig 2B) significantly increased the relative miR-1 (p<0.05) and miR-133a (p<0.05) expression in the HH group compared with the NC group. Electrical stimulation not only prevented the increase in miR-1(p<0.05) but also significantly decreased the miR-133a expression in HE group compared with the HH group (p<0.05). However, the miR-1 expression in the HE group remained increased compared with the NC group (p<0.05). A strong reduction in the miR-133b expression was also identified in the HE group because there was no significant difference between the groups after 4 weeks.

### 3. Changes in miR-1-related protein expression

**Fig 3:**
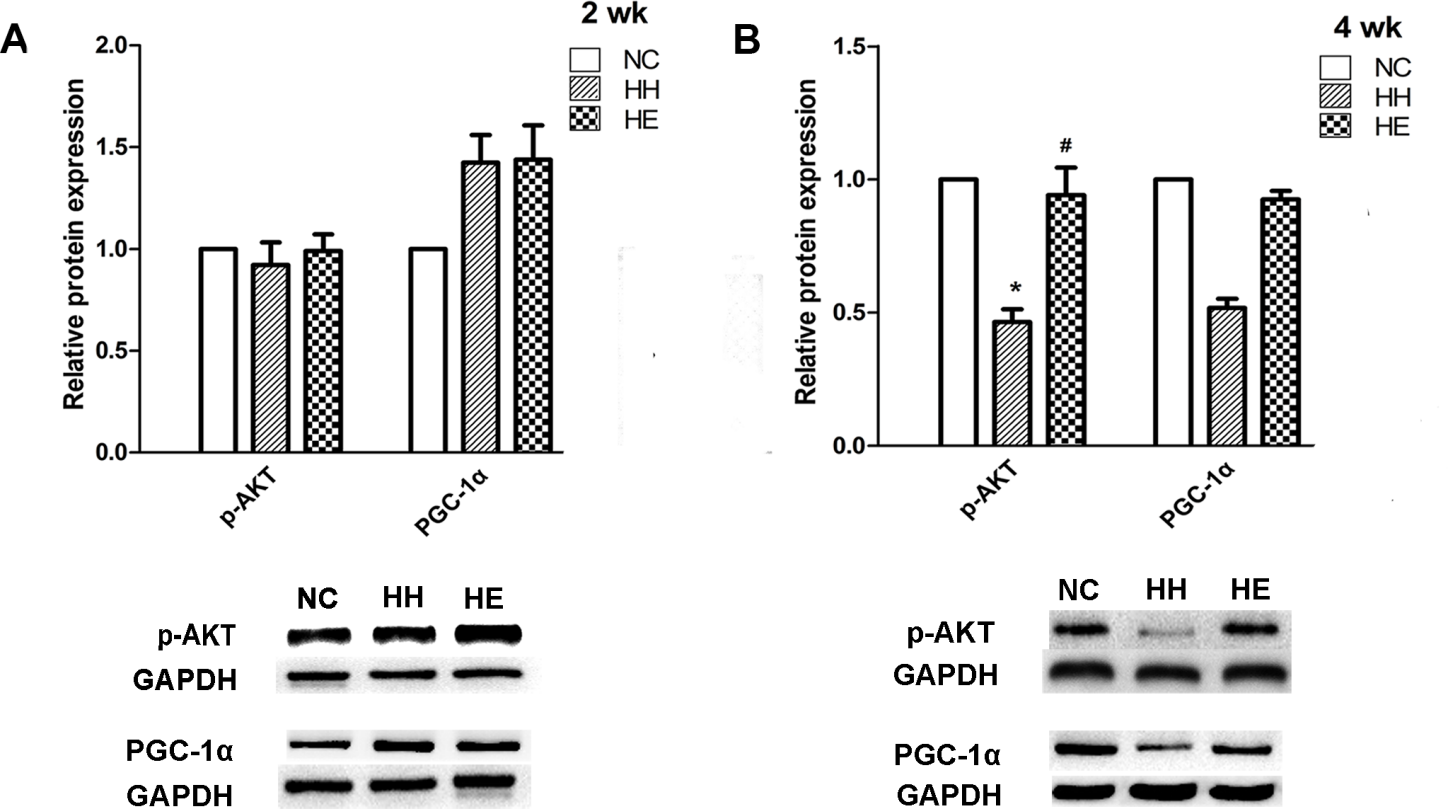
Effects of electrical stimulation on the CIHH-induced reduction of PGC-1α and p-Akt protein. Gastrocnemius muscles were isolated from the lower limb of three groups of rats at 2 weeks (A) and 4 weeks (B). Analyses of the expression of PGC-1α and p-Akt proteins were performed by Western blotting. Tubulin and GAPDH were used as loading controls. The optical density values were normalized to their respective Tubulin and GAPDH loading controls, and the means±SEMs were graphed (relative expression) to semi-quantitatively compare the protein levels. *p<0.05 (at least) vs. NC group; #p<0.05 (at least) vs. HH group. NC = normal control group; HH = hypoxia-hypercapnia group; HE = hypoxia-hypercapnia + electrical stimulation.

**Fig 4:**
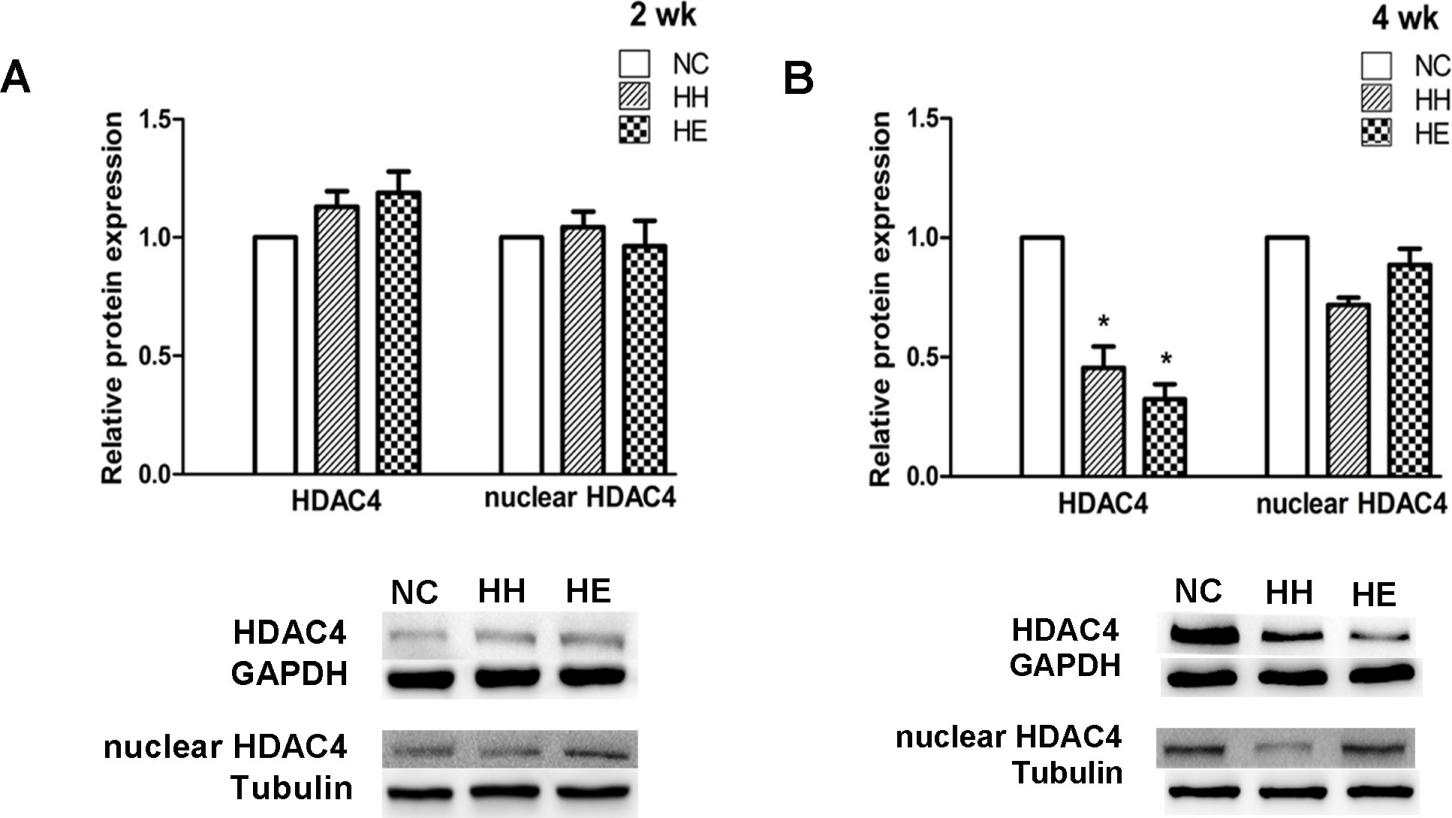
Effects of electrical stimulation on the CIHH-induced reduction of HDAC4 and nuclear HDAC4 protein. Gastrocnemius muscles were isolated from the lower limb of three groups of rats at 2 weeks (A) and 4 weeks (B). Analyses of the expression of HDAC4 and nuclear HDAC4 proteins were performed by Western blotting. Tubulin and GAPDH were used as loading controls. The optical density values were normalized to their respective Tubulin and GAPDH loading controls, and the means±SEMs were graphed (relative expression) to semi-quantitatively compare the protein levels. *p<0.05 (at least) vs. NC group; #p<0.05 (at least) vs. HH group. NC = normal control group; HH = hypoxia-hypercapnia group; HE = hypoxia-hypercapnia + electrical stimulation.

IGF-1 is one target of miR-1; therefore, we analyzed the activity of the IGF-1 pathway by examining the p-AKT[14]. We also determined the expression of HDAC4 and PGC-1α, which are involved in the regulation of the another target pathway of miR-1[13]; the nuclear HDAC4 protein was assessed to measure the deacetylase activity of HDAC4, which controls its subcellular localization[24]. Western blotting was performed to determine changes in the expression levels of these proteins. After 2 weeks, the expression of PGC-1α, p-AKT (Fig 3), HDAC4 and nuclear HDAC4 proteins (Fig 4) was no significant difference between the groups; however, there was a tendency towards an increase in the PGC-1α protein expression in the HH and HE groups. After 4 weeks, significant reductions were identified in the content of the p-AKT and HDAC4 proteins in the HH group compared with the NC group (p<0.05). The reduction in the expression of PGC-1α and nuclear HDAC4 proteins in the HH group also exhibited a strong downward trend (p = 0.051 and p = 0.058, respectively). Electrical stimulation significantly suppressed the altered expression levels of p-AKT (p<0.05) and PGC-1α (p = 0.053) in the HE group compared with the HH group (Fig 3). Surprisingly, the HDAC4 protein (Fig 4) in the HE group did not reversed by electrical stimulation was even more decreased compared with the NC group (p<0.01). In contrast, the nuclear HDAC4 protein (Fig 4) in the HE group was increased compared with the HH group; although this change was not significant, it was a notable phenomenon.

### 4. Changes in miR-133a/b-related protein expression

**Fig 5:**
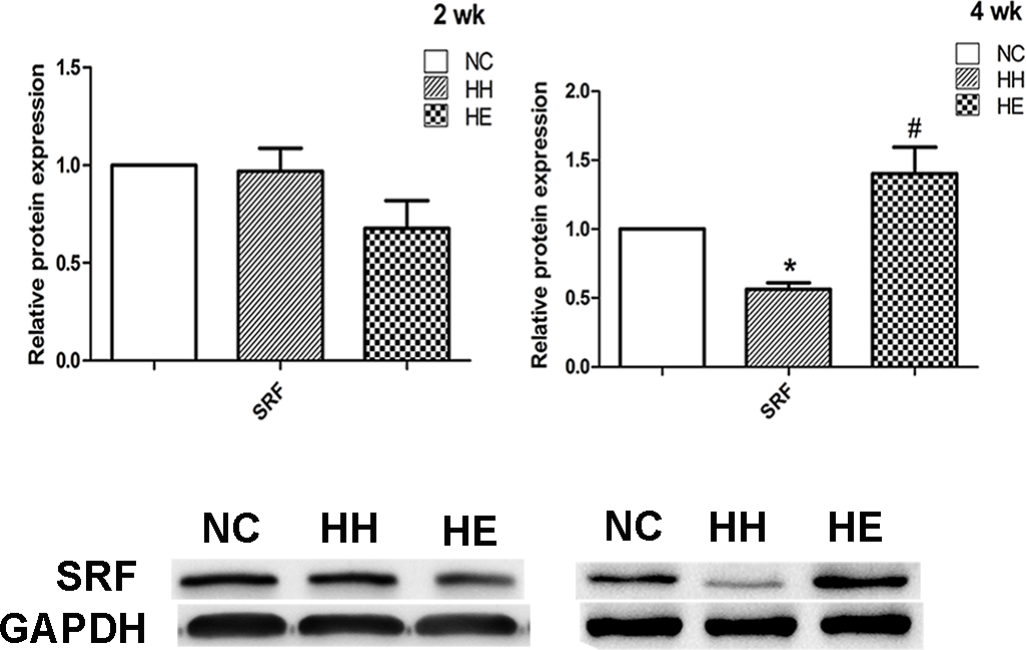
Effects of electrical stimulation on the CIHH-induced reduction of protein expression of SRF. Gastrocnemius muscles were isolated from the lower limb of three groups of rats. Analyses of the SRF protein expression were performed via Western blotting at 2 weeks and 4 weeks, and GAPDH was used as a loading control. The optical density values were normalized to their respective Tubulin and GAPDH loading controls, and the means±SEMs were graphed (relative expression) to semi-quantitatively compare the protein levels. *p<0.05 (at least) vs. NC group; #p<0.05 (at least) vs. HH group. NC = normal control group; HH = hypoxia-hypercapnia group; HE = hypoxia-hypercapnia + electrical stimulation.

**Fig 6:**
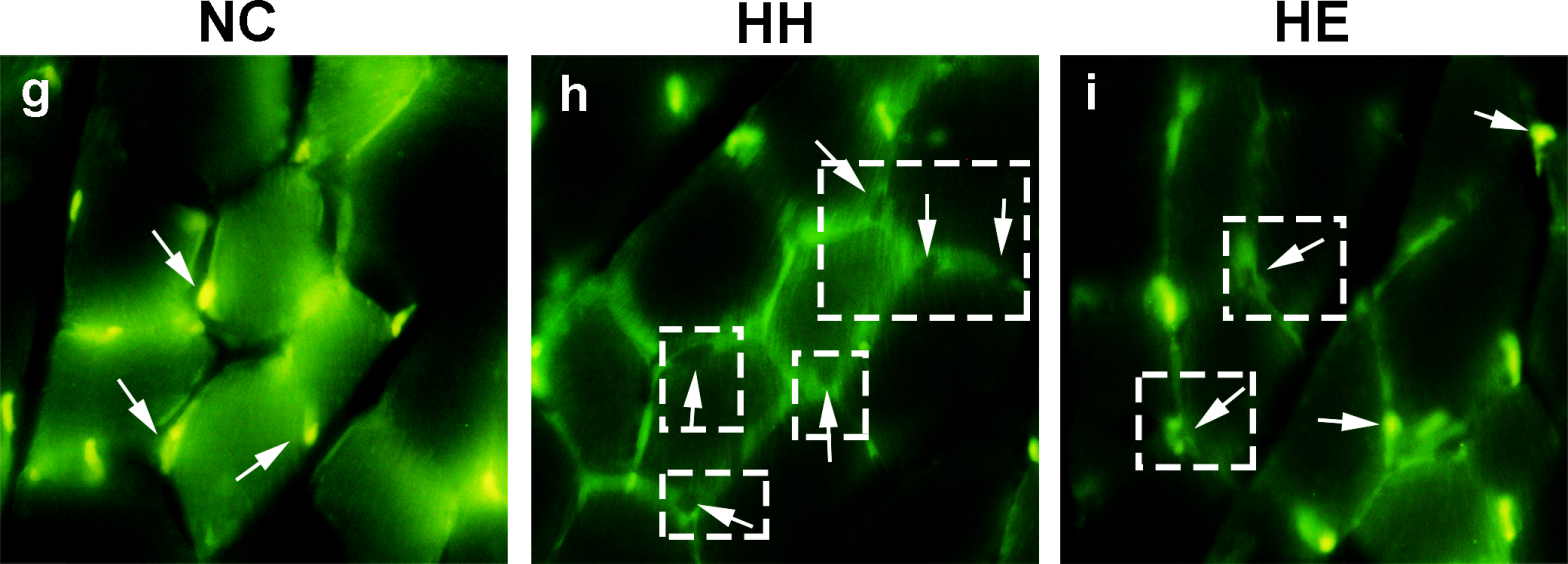
Effects of electrical stimulation on the CIHH-induced nuclear localization change of SRF (4 weeks). Gastrocnemius muscles were isolated from the lower limb of three groups of rats. Nuclear localization of SRF was determined by immunofluorescence staining; observations via confocal microscope indicate the localization of SRF in green fluorescence and the nuclei with 4,6-diamidino-2-phenylindole (DAPI) in blue fluorescence as a comparison(see S1 Fig). Arrowheads show the localization of nuclei staining and the inset shows perinuclear staining for SRF. NC group showed that the staining pattern of the factor was identified in the nucleus as well as in the cytoplasm (g). After 4 weeks, some parts of HH group showed exclusively cytoplasmic in HH group (h). In HE group nuclear localization change of SRF was partly reversed (i). Detailed images of both 2 weeks and 4 weeks are shown in S1 Fig. Original magnification 40×. NC = normal control group; HH = hypoxia-hypercapnia group; HE = hypoxia-hypercapnia + electrical stimulation.

miR-133 (including miR-133a and miR-133b) regulates muscle proliferation through SRF[13]; thus, we analyzed the expression of SRF in the gastrocnemius via Western blotting (Fig 5). SRF activity is also regulated by the localization of the protein, which was determined by immunofluorescence (Fig 6)[13]. The SRF protein at 2 weeks was not significantly different between the groups, after 4 weeks CIHH exposure, the SRF expression was decreased (p<0.05); and it was more decreased (p<0.01) by electrical stimulation (Fig 5). In the other hand, after 2 weeks of CIHH exposure, the nuclear staining of SRF was not significantly different between the groups (S1 Fig). After 4 weeks, SRF was easily detectable in the nuclei of the NC group (Fig 6). However, there was a reduction in the nuclear SRF staining of the HH group (p<0.05) and the appearance of perinuclear SRF staining in the HH group. Moreover, electrical stimulation led to increased nuclear staining in the HE group compared with the HH group (p<0.05).

## Discussion

Skeletal muscle dysfunction in COPD has been widely acknowledged; however, the exact pathogenesis remains controversial, inactivity appears to be a major contributor to skeletal muscle dysfunction in COPD because changes in lower limb muscles, such as muscle atrophy and fiber type shift present in COPD, are the typical physiological responses to muscle disuse/inactivity[25]. Furthermore, the characteristic metabolic derangements in COPD muscle dysfunction can be explained by muscle atrophy and fiber type shift[7].

The p-AKT related signaling pathway have been associated with muscle atrophy[26], which is involved in the regulation of the quadriceps muscle mass in patients with COPD[27]. In our study, the p-AKT expression was down-regulated in the HH group compared with the NC group.This indicates that muscle atrophy may occurred in rats after CIHH. miR-1 has an important role in the mediation of the IGF-1 pathway, which comprises a negative feedback loop between miR-1 expression and the IGF-1 signal transduction cascade[14]. It may explain the observation from our study that significantly increased miR-1 expression accompanied reduced p-AKT protein expression in the HH group. However, these changes was reversed by electrical stimulation,in HE group, miR-1 expression was reduced and p-AKT expression was increased compared with HH group. Electrical stimulation exhibits a positive role in protecting muscle from atrophy.

PGC-1α is a principal factor that regulates muscle fiber type determination and is involved in mitogenesis, antioxidant system regulation and differentiation to type I fiber[28]. HDAC4 is one of the main targets of miR-1 in the regulation of muscle differentiation[13, 29]. HDAC4 have a intimate connection with PGC-1α via myocyte enhancer factor-2 (MEF-2) which is a muscle-enriched transcriptional factor thought to be the foremost characterized partner of HDAC4[28, 30–32]. It has been reported that the PGC-1α related signaling pathway in the skeletal muscle of COPD patients is decreased compared with controls[33]. In our study, the PGC-1α expression was reduced in the HH group, which was accompanied by muscle fiber shift (Ⅱa→Ⅱb). We also identified a reduction in HDAC4 protein in the gastrocnemius of the HH group, as well as the location of HDAC4 in the nucleus. On the other hand, the miR-1 expression was extremely high in HH group. miR-1 has been demonstrated to suppress HDAC4 expression in the skeletal myoblast[29], which may explain the increased expression of miR-1 with the lower HDAC4 protein level in the HH group compared with the NC group. In our study, HDAC4 expression was opposite to skeletal muscle dysfunction, in other words, The HDAC4 may function as a protective factor for muscle which was also supported by other two researchs[24, 34].

After 4 weeks electrical stimulation, the HDAC4 protein reduction was enhanced in HE group compared with HH group, on the contrary, the location of HDAC4 in the nucleus was increased (although it did not reach statistical significance). It suggested that HDAC4 exhibited nuclear influx in the HE group, and according to other research the beta-adrenergic signaling pathway to HDAC4 (beta-adrenergic receptor → cAMP → PKA → HDAC4 phosphorylation at the PKA sites) may play a role in such process[35]. Combined with the previously discussed observations, we may think that the nuclear location of HDAC4 plays a more positive role in CIHH induce rat muscle dysfunction than the total HDAC4 protein.

miR-133 enhances myoblast proliferation by repressing SRF, which is a transcription factor known to block cell proliferation[13]. Previous research has demonstrated that reduced MRTF/SRF axis activity contributed to slow-to-fast muscle fiber shift[5]. Thus, in our study, reduced SRF expression and activity in the HH group may suggest a fast muscle phenotype change. Interestingly, only miR-133a exhibited a significant increase in the HH group at both 2 and 4 weeks, and partly reversed by 4 weeks electrical stimulation. miR-133b expression exhibited a temporary increase after 2 weeks of electrical stimulation in the HE group. Furthermore, SRF was significantly reduced in HH group, and followed by a significant increase after 4 weeks electrical stimulation in HE group. From our study, there is a fact that although miR-133a and miR-133b have very similar sequences[13], they still have distinct functions for muscle specific expression[36]. miR-133a may represent a better reflection of CIHH induce rat muscle dysfunction than miR-133b.

In the traditional view, low-frequency (e.g., 10 HZ), high-volume (i.e., 4 h/day, 7 days/wk) electrical stimulation may promote mitochondrial biogenesis and a fast-to-slow muscle fiber phenotype transformation. In contrast, high-frequency (e.g., 100 HZ), low-volume (i.e., 6x10 repetitions of 3 s-bursts) electrical stimulation may promote hypertrophy via the stimulation of muscle protein synthesis[37]. However, a previous research report indicated that an 8 weeks electrical stimulation with a 75 Hz intermittent pattern is characteristic of both resistance (i.e., strength gains) and endurance (i.e., fast-to-slow and glycolyticto-oxidative conversion) training[18]. This finding may primarily be ascribed to the nonselective, continuous, and synchronous motor unit recruitment pattern[18]. In our study, 4 weeks of electrical stimulation with 100 Hz-2 Hz intermittent pattern also induces atypical adaptations in rat muscles. Even more important, these adaptations may relieve CIHH induced muscle atrophy and muscle fiber type shift through miRNAs to impact the PGC-1α, HDAC4, p-AKT and SRF signaling pathways.

## Conclusion

The findings presented here demonstrate that the CIHH rats exhibited a significant reduced running capacity and prominent slow-to-fast muscle fiber shift, which was accompanied by significant increased in miR-1, miR-133 expression and significant reduced in PGC-1α, HDAC4, p-AKT and SRF expression. Chronic electrical stimulation exhibits a protective role in the alleviation of CIHH-induced muscle impairment by reversing these changes. It is reasonable to speculate a priority that miR-1 and miR-133 may have roles in the response of CIHH-impaired muscle to changes during electrical stimulation via regulation of related signaling pathways.

## Supporting Information

S1 Fig Effects of electrical stimulation on the CIHH-induced nuclear localization change of SRF (2 weeks and 4 weeks). (TIF)
