# Supplementary figures and images for: Electrical Stimulation Improves Rat Muscle Dysfunction Caused by Chronic Intermittent Hypoxia-Hypercapnia via Regulation of miRNA-Related Signaling Pathways
Source: PLoS One. 2016 Mar 29;11(3):e0152525. doi: 10.1371/journal.pone.0152525 (PMC4811440; doi:10.1371/journal.pone.0152525)

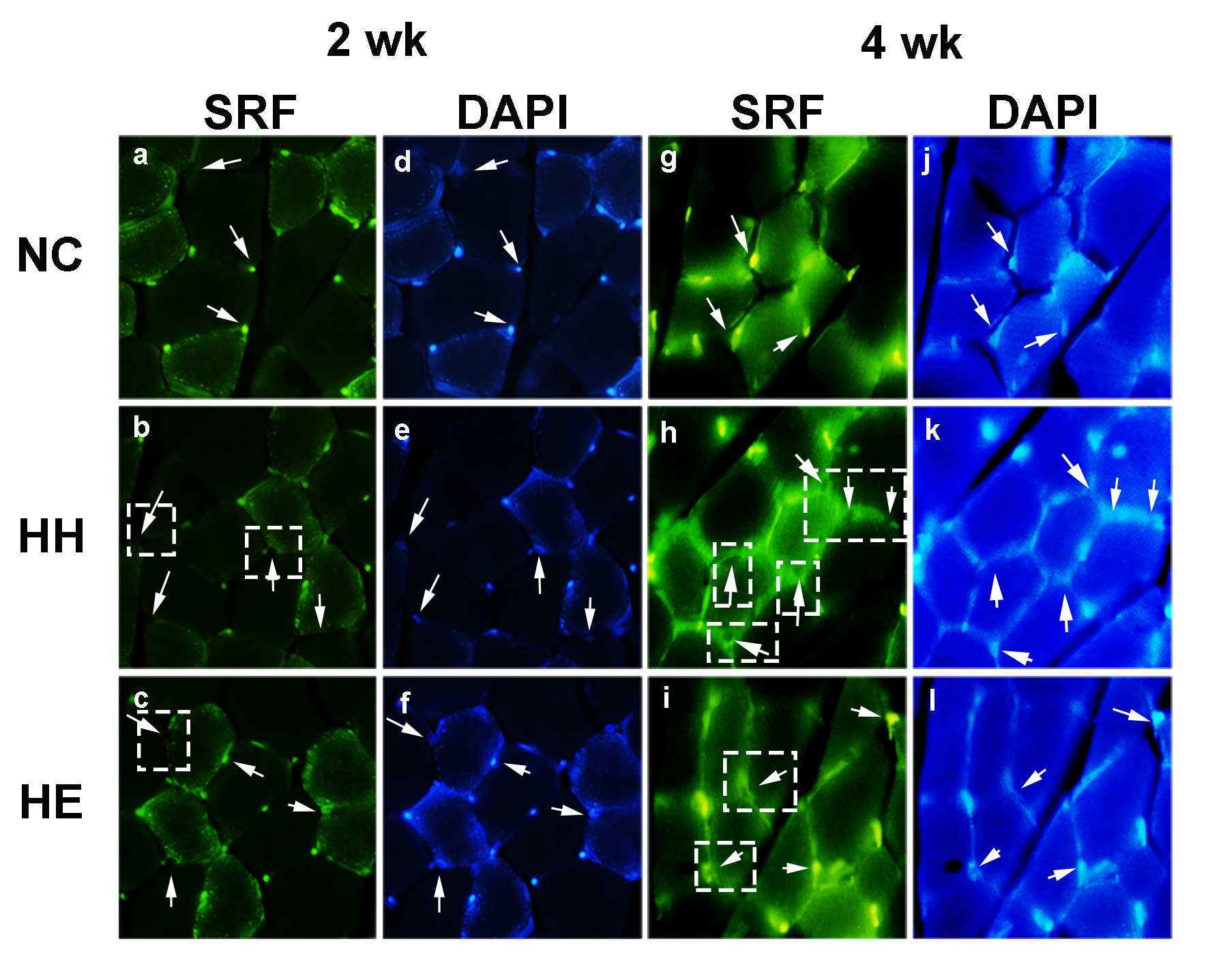

Supplement: S1 Fig — (TIF) [file pone.0152525.s001.tif]
